# Alternative Approaches in Gene Discovery and Characterization in Alzheimer’s Disease

**DOI:** 10.1007/s40142-013-0007-5

**Published:** 2013-01-22

**Authors:** Nilüfer Ertekin-Taner, Phillip L. De Jager, Lei Yu, David A. Bennett

**Affiliations:** 1 Departments of Neurology and Neuroscience, Mayo Clinic Florida, 4500 San Pablo Road, Birdsall 3, Jacksonville, FL 32224 USA; 2 Departments of Neurology and Psychiatry, Program in Translational NeuroPsychiatric Genomics, Institute for the Neurosciences, Brigham and Women’s Hospital, 77 Avenue Louis Pasteur NRB168, Boston, MA 02115 USA; 3 Harvard Medical School, Boston, MA 02115 USA; 4 Program in Medical and Population Genetics, Broad Institute, 7 Cambridge Center, Cambridge, MA 02142 USA; 5 Rush Alzheimer’s Disease Center, Rush University Medical Center, Chicago, IL 60612 USA

**Keywords:** Alzheimer’s disease, Endophenotype, Gene expression, Neuropathology, Cognition, Genetics

## Abstract

Uncovering the genetic risk and protective factors for complex diseases is of fundamental importance for advancing therapeutic and biomarker discoveries. This endeavor is particularly challenging for neuropsychiatric diseases where diagnoses predominantly rely on the clinical presentation, which may be heterogeneous, possibly due to the heterogeneity of the underlying genetic susceptibility factors and environmental exposures. Although genome-wide association studies of various neuropsychiatric diseases have recently identified susceptibility loci, there likely remain additional genetic risk factors that underlie the liability to these conditions. Furthermore, identification and characterization of the causal risk variant(s) in each of these novel susceptibility loci constitute a formidable task, particularly in the absence of any prior knowledge about their function or mechanism of action. Biologically relevant, quantitative phenotypes, i.e., endophenotypes, provide a powerful alternative to the more traditional, binary disease phenotypes in the discovery and characterization of susceptibility genes for neuropsychiatric conditions. In this review, we focus on Alzheimer’s disease (AD) as a model neuropsychiatric disease and provide a synopsis of the recent literature on the use of endophenotypes in AD genetics. We highlight gene expression, neuropathology and cognitive endophenotypes in AD, with examples demonstrating the utility of these alternative approaches in the discovery of novel susceptibility genes and pathways. In addition, we discuss how these avenues generate testable hypothesis about the pathophysiology of genetic factors that have far-reaching implications for therapies.

## Introduction

**Fig. 1:**
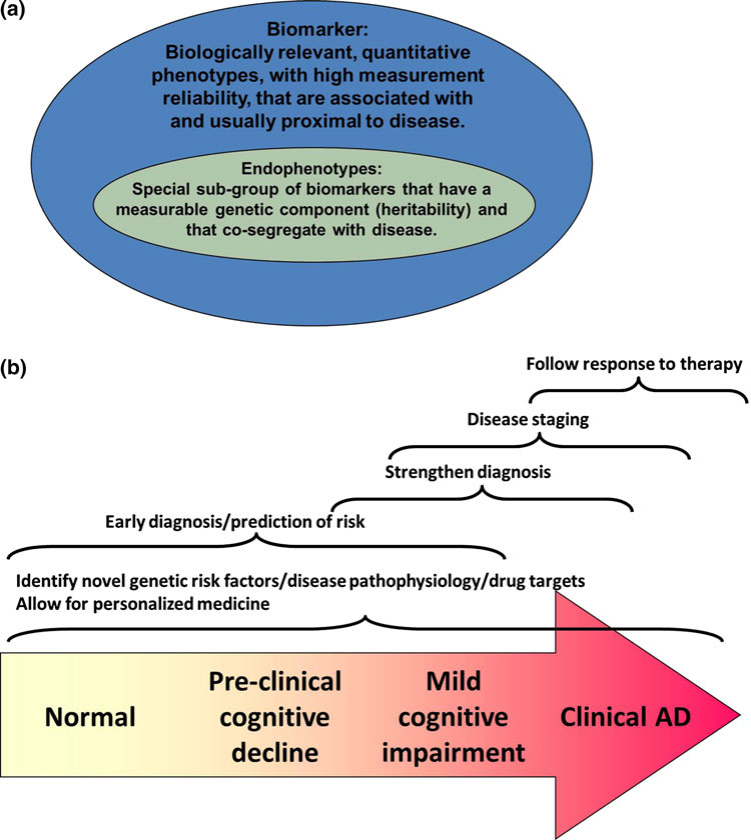
Definition (**a**) and utility (**b**) of endophenotypes. The *graded arrow* represents the continuum of AD with *darker colors* symbolizing greater clinical expression of disease. The *brackets* and *text* above them depict the different uses of endophenotypes at various disease stages (Color figure online)

Genetic studies of human diseases have been marked by an explosion in the number of susceptibility loci identified through genome wide association studies (GWAS) in the past several years. Similar to other complex disorders, neuropsychiatric diseases, too, had a share of their genetic risk loci discoveries, with—for example—28 published studies to date on Alzheimer’s disease (AD), 18 on bipolar disorder and 22 on schizophrenia, according to the Catalog of Published GWAS accessed on 21 Aug 2012 [1]. The translation of this success to viable therapies and biomarker discovery depends on the identification and characterization of the actual disease genes and functional variants at these susceptibility loci. Furthermore, despite the large number of discovered loci, a substantial component of genetic susceptibility remains unexplained for complex diseases [2•]. To overcome these major hurdles in the post-GWAS era requires a multitude of alternative approaches including the use of endophenotypes, which are biologically-relevant, quantitative and heritable phenotypes [3•] (Fig. 1a).

The endophenotype approach was initially advocated in psychiatric genetics [4, 5] due to the need to have an objective and quantifiable outcome in genetic studies, given the relatively imprecise nature of the clinical diagnosis, which is thought to result in heterogeneity. The rationale for using endophenotypes, instead of or in addition to the binary disease phenotypes, stems from the following assumptions: (1) Endophenotypes represent an intermediate outcome between genes and clinical diagnosis of a disease, and given their closer proximity to the genetic variation than the disease outcome, the genetic component influencing the endophenotype will be larger and therefore easier to detect [6, 7]; (2) Endophenotypes are under the influence of a smaller number of genes than the more complex disease outcome [6]; (3) Given its quantitative nature, using an endophenotype as an outcome variable will be statistically more powerful than the binary case/control approach in detecting genetic associations; (4) Since they are objectively quantifiable, endophenotypes constitute a more homogeneous and accurately measurable phenotype than disease outcome; (5) The endophenotype approach can allow inclusion of individuals with and without a given diagnosis, which will increase power, particularly in family studies or for traits that are age dependent [7, 8]; (6) The endophenotype approach will provide information about the underlying mechanism of action for the gene and variant of interest and might therefore more readily enable downstream functional investigations, including the generation of animal models with quantifiable outcomes [9]. While the accuracy of these assumptions needs to be established, the endophenotype approach has begun to generate hypotheses for novel genetic loci and pathways implicated in human disease and to enable the downstream characterization of disease variants and genes, as exemplified in this review (Fig. 1b).

AD, the most common dementia in the elderly, is especially amenable to the endophenotype approach, for a number of reasons. First, AD has a distinct neuropathology characterized by accumulation of amyloid β (Aβ) in senile plaques and hyperphosphorylated tau in neurofibrillary tangles, both of which are quantifiable phenotypes, and the latter correlates with clinical disease severity [10]. Second, discoveries of Mendelian mutations in the amyloid precursor protein (*APP*), presenilin 1 (*PSEN1*) and *PSEN2* genes in early-onset AD that lead to elevations in secreted Aβ and their modeling in animals harboring these mutations (reviewed [11]) bolstered the amyloid cascade hypothesis [12]. The ability to measure Aβ levels in the serum and cerebrospinal fluid (CSF) of AD patients and their relatives [13], and the determination that Aβ levels are heritable [14] enabled the first studies utilizing Aβ levels as endophenotypes in genetic studies that discovered genetic loci and variants influencing AD risk and Aβ [8, 15–17]. This was followed by investigations of CSF Aβ [18] and tau levels [19] as endophenotypes in AD genetics studies. Third, the availability of prospective, elderly cohorts with rich clinical, neurocognitive and neuroimaging measures [20–22], knowledge that many of these measures are heritable [6, 7, 23], and detection of preclinical changes in these measures (reviewed [24••]) advocate their use as endophenotypes in genetic studies of AD. Fourth, the advent of technology that allows measurement of gene expression levels for all known transcripts (transcriptome), development of methodologies that allow analysis of this data at the whole-genome level, significant heritability attributed to gene expression levels (reviewed [3•]) and the availability of well-characterized brain tissue from neuropathologic AD and other patients in which transcriptome can be measured, empowered the use of gene expression levels from brain and other tissues as endophenotypes in AD.

This review focuses on three types of endophenotypes in AD: gene expression levels, neuropathologic measures and cognitive measures. These diverse endophenotypes span the vast spectrum of biological insights that can be gained by this quantitative approach: genetic associations with transcript levels, the most proximal of these traits to the susceptibility allele, may uncover the initial mechanism for the functional consequences of the allele. Neuropathologic phenotypes can relate a variant to the known neuropathology of the disease and might enable the dissection of pathophysiologic pathways influenced by the polymorphism of interest. Finally, the use of cognitive endophenotypes can uncover genetic risk factors governing distinct aspects of human cognition and the clinical expression of the disease. We recognize that there are many other endophenotypes that are currently utilized or are excellent candidates for genetic studies of AD, including Aβ and tau levels, neuroimaging measures such as hippocampal volume and magnetic resonance spectroscopy levels and methylation patterns. Although a comprehensive assessment of all these endophenotypes is beyond the scope of this review, the generalizations that can be drawn from this synopsis could potentially be applicable to many other quantitative phenotypes in AD research.

## Gene Expression Endophenotype

Gene expression levels constitute a special group of endophenotypes for a number of reasons. First, because the tested phenotype is the level of the expressed gene transcript(s), genetic studies of gene expression endophenotypes (also known as expression quantitative trait loci or eQTL studies) directly identify the gene under the influence of genetic variants. This is in contrast to any other phenotype, where genetic studies merely implicate a “locus of interest” without definitive information about the affected gene. This first characteristic of the gene expression endophenotype can be utilized to uncover plausible disease genes via combined assessment of gene expression endophenotype and disease phenotype as discussed below. Second, genetic factors identified via eQTL studies provide guidance about the underlying mechanism of action of the “functional variants” at the “locus of interest”. This can enable a more directed search for such “functional variants”, for example by focusing on regulatory variants that influence whole transcript levels or splice isoforms. Furthermore, such information can guide downstream in vitro studies that are more relevant to the underlying genetic variation. Third, the ability to assess concerted expression level changes or eQTL associations at the transcriptome level via pathway analysis can lead to identification of novel biological networks that may underlie disease pathophysiology. Below, we discuss the utility of gene expression endophenotypes in gene discovery and characterization in AD, highlighting examples that take advantage of these special characteristics of this approach.

### Utility in Gene Discovery

The utilization of gene expression endophenotypes in gene discovery in AD first began with transcriptome profiling (or mRNA profiling) studies, which are recently comprehensively reviewed [25]. The underlying premise of these studies is that mRNA from patients with disease will have changes in comparison to controls; and that these changes may underlie disease pathophysiology. The most important caveat in this assumption is that the detected gene expression changes may be a consequence of the disease and non-specific, rather than a causal event [3•]. This pitfall is especially concerning if the transcriptome profiling is performed in tissue affected by the disease (such as the temporal cortex in AD). Indeed, in a detailed microarray-based transcriptome profiling study of 14 different cerebral cortex regions and the hippocampus, from 69 autopsied AD subjects of varying clinical and pathologic severity versus 18 controls (maximal number of subjects utilized in the study), Haroutunian et al. [26] identified the greatest number of gene expression changes in regions from the temporal cortex across the disease stages, with increasing changes occurring in later disease stages and stronger correlations between gene expression and disease severity seen in more advanced disease. Importantly, most of the changes observed were downregulations rather than upregulations. Collectively, these results could imply that the progression of disease and cell loss may be driving these changes, rather than vice versa. These authors [26] and others [27] attempted to overcome this concern by analysis of autopsied AD subjects with mild neuropathology and concluded that gene expression changes that occur in regions prior to the development of neuropathology are unlikely to be a consequence of the disease process. Bossers et al. [27] analyzed 49 prefrontal cortex samples from subjects with Alzheimer’s-type neuropathology to identify correlated changes in gene expression which varied with advancing Braak stage. They determined that the most significant changes occurred in “synaptic activity genes” between Braak stages II and III, which is prior to or just at the onset of AD-type neuropathology when the subjects were clinically non-demented. The authors also noted that levels of several genes correlated with increasing intracellular Aβ levels during these Braak stages, leading them to postulate that expression changes in genes of synaptic activity may be a coping mechanism against increased Aβ that occurs prior to clinical and neuropathological AD. While it is not possible to draw definitive conclusions about the longitudinal cascade of events, including gene expression changes, based on cross-sectional assessment of brain tissue from small numbers of distinct subjects, these results nevertheless generate intriguing hypotheses about AD pathophysiology via correlative analysis of transcriptome and neuropathology data.

Another approach in utilizing gene expression endophenotypes in gene discovery is combined transcriptome profiling and AD risk association studies. In a small hippocampal mRNA profiling study of six AD versus two control brains, Li et al. [15] detected lower *GSTO1* (glutathione *S*-transferase omega-1) levels in the AD brains, followed by significant associations with age-at-onset of both AD and Parkinson’s disease with variants in both *GSTO1* and its nearby homologue *GSTO2* [28]. This prompted follow-up genetic studies with disease risk and/or age-at-onset phenotypes with mixed results [29–33]. In a GWAS of brain gene expression (brain eGWAS) levels in ~800 tissue samples from ~400 brains [34•], we identified strong associations with variants at this locus and brain *GSTO2* but not *GSTO1* levels [35], consistent with results from another brain eQTL study [36]. In our study, we determined that the same variant associated with both lower brain levels of *GSTO2* as well as increased AD risk in older subjects, which is biologically consistent with the antioxidant functions of this gene. Furthermore, pathway analysis of the significant genes in our brain eGWAS showed significant enrichment for glutathione metabolism genes, suggesting there may be additional genes in this pathway with potential influence on AD and other neurodegenerative diseases. Other genes which were detected by expression profiling studies of AD versus control tissue, followed by significant associations with AD risk, include *POU2F1* [37] and *IL*-*33* [38]. These studies highlight the potential utility of the gene expression endophenotype in identifying gene(s) and pathways that may harbor regulatory variants that influence disease risk.

More recently, joint assessment of disease GWAS with eQTL studies have been advocated to prioritize suggestive results from disease GWAS and/or identify novel candidate disease genes, based on the premise that disease variants will be enriched for regulatory variants that influence gene expression and vice versa [39••]. Indeed, in a comparison of eQTL results from lymphoblastoid cell lines from HapMap samples with human disease/trait GWAS summary data, Nicolae et al. [40] identified significant enrichment for SNPs that influence expression (eSNPs) amongst human disease/trait associating variants. Combined assessment of brain expression endophenotype associations [36] with disease GWAS showed enrichment for eQTLs amongst schizophrenia risk alleles [41]. This approach, combined with pathway analysis led to nomination of novel genes for diabetes in another study [42]. We have applied this approach for the first time to a large AD GWAS [43], by combining with our brain eGWAS data and detected an enrichment for significant eSNPs amongst suggestive AD risk SNPs [34•]. These results suggest that AD, like other complex human diseases, may at least in part be influenced by regulatory variants. The novel genes detected in the “grey zone” of disease GWAS by this approach warrant further studies for identification of functional variants and to demonstrate their downstream regulatory effects.

### Utility in Gene Characterization

Gene expression endophenotypes can also be used to characterize the effects of disease risk variants and their downstream consequences on the disease gene. A prime example of this is *MAPT*, which has rare variants leading to frontotemporal dementia with parkinsonism linked to chromosome 17, as well as common variants within a haplotype block that associate with multiple taupathies (reviewed [44]). These variants have been shown to influence either splicing or transcriptional activity of *MAPT* [45–48].

Another example of gene expression endophenotype explorations for a known risk gene is APOE, which has common missense polymorphisms, leading to three isoforms *APOE* ε2, ε3 or ε4, where *APOE* ε4 has clearly been shown to influence AD risk, whereas *APOE* ε2 might confer protection from AD [11, 49]. APOE isoforms have dual types of functions in the brain with roles in both maintaining neural health and also in promoting AD pathophysiology (reviewed [50]). In addition to the most well-studied isoforms, a number of promoter region polymorphisms have been identified for *APOE* that impart risk for AD, at least partially independently of the *APOE* isoform (reviewed [51]). Although consensus is still lacking, the most well studied promoter region polymorphism −491AA appears to confer AD risk independent of *APOE* ε4 and increase *APOE* transcriptional activity [52, 53], suggesting that both *APOE* isoforms and levels may play a role in AD pathogenicity. Despite absence of conclusive evidence for the role of the promoter region polymorphisms in AD risk, the transcriptional complexity of *APOE* and its dual role in the central nervous system (CNS), *APOE*-directed therapeutics aimed at modifying its levels are advocated for treatment of AD. A recent study in animal models of AD demonstrated clearing of Aβ and reversal of behavioral and electrophysiologic deficits upon treatment with a transcriptional inducer of *APOE* [54].

It is similarly critical to characterize the novel AD candidate variants and genes that are being identified in late-onset AD (LOAD) GWAS (reviewed [55]) with respect to their influence on gene expression endophenotypes. This information will provide focus for the downstream functional variant discovery, in vitro and in vivo studies and ultimately set the stage for the search of therapeutics targeting the appropriate mechanisms and pathways. We have begun to characterize the novel LOAD GWAS variants for their influence on gene expression endophenotypes using our brain eGWAS [56•] and eQTL analyses of data generated from peripheral immune cells [57]. The latter study demonstrated that AD-associated variants, such as the one in the *PICALM* locus, influence gene expression in non-resident CNS cells and suggest that infiltrating immune cells may play a role in the onset of AD. On the other hand, the brain data identified association between the top AD risk variants at the *CLU* and *MS4A* loci with brain levels of *CLU* and *MS4A4A* genes, implicating regulatory genetic variation for these genes in AD risk. Furthermore, we detected additional strong gene expression associations for both *CLU* and *ABCA7*, some of which also confer AD risk, independent of the top GWAS variants, suggesting that new regulatory AD variants might exist at these loci, in addition to the top SNPs already identified by disease GWAS. Our findings in *CLU* are corroborated by Ling et al. [58•] who determined that the AD-protective *CLU* variant is also associated with higher CLU1 isoform levels in human brains. The direction of the gene expression endophenotype effect is identical in these two studies, and indicate that therapeutic approaches aimed at increasing levels of CLU in the brain might confer protection from AD. Interestingly, valproic acid (VPA), a well-known anti-epileptic and anti-depressant with histone deacetylase (HDAC) inhibiting properties, was shown to induce *CLU* expression in astrocytes [59]. VPA was also previously highlighted as a potentially promising drug for AD due to its pro-neurogenesis and neuroprotective properties [60]. Although clinical trials of VPA in AD patients have yet failed to demonstrate a beneficial outcome [61, 62], evidence warrants further investigations along the CLU induction axis as a potential therapeutic avenue in AD. Gene expression endophenotypes may be informative biomarkers in such future therapeutic trials.

## Neuropathology Endophenotype

Despite recent achievements by various GWAS consortia [43, 63–66], a large proportion of the genetic contribution to Alzheimer disease still remains to be identified. The utility of the conventional approach that relies heavily on clinical diagnosis (i.e., a dichotomy of cases vs. controls) is dampened by contamination of the control group with persons with pre- or sub-clinical disease. Use of the intermediate neuropathologic endophenotype helps to address these disadvantages. While the majority of persons clinically diagnosed with AD have AD pathology [67–69], AD pathology is also common among persons without dementia [70–73]. Neuropathologic abnormality have been reported in persons both with and without cognitive impairment [74, 75], suggestive of a disease process involving pathologic change of brain structure. Moreover, the phenotypic heterogeneity of dementia reflects a broader spectrum of neurodegenerative conditions other than AD, including cerebrovascular infarctions, neocortical Lewy bodies, and TAR DNA-binding protein 43 (TDP-43), just to name a few. Each of these diseases independently contributes to the clinical dementia phenotype [76, 77]. Further, this heterogeneity extends to the probable AD phenotype [78]. Given this context, neuropathologic phenotypes provide several important implications.

First, compared to the more distal clinical phenotypes, neuropathologic traits lie directly in the pathway connecting genetic actions to the clinical expression of AD dementia. In other words, genetic variants do not directly cause cognitive decline and AD, but rather contribute to a series of events associated with neuropathology; these, in turn, result in cognitive decline and AD. Thus, utilization of neuropathologic outcomes increases statistical power to discover genetic variants that influence AD-related processes. Using the well-known apolipoprotein E genotype (*APOE*), we demonstrated that among a group of only about 500 community based elderly with European ancestry, quantitative pathologic AD phenotypes provide considerably more power than phenotypes of clinical AD diagnosis or cognitive function [79•]. In this analysis, the association of the protective *APOE* ε2 allele with clinical AD and level of cognition were not significant. However, it has a strong association with a measure of overall burden of AD pathology (*p* = 10 × 10^−5^). Similar differences were seen with the ε4 allele. We subsequently showed that measures of AD pathology mediated the association of allele status with cognitive decline illustrating that AD pathology is in the causal chain linking the genetic variant with cognitive decline [80]. Other studies have reported similar findings [81].

Second, genetic associations with clinical outcomes are confounded by misclassification of pre- and sub-clinical subjects. These are people that are harboring genetic variants that link to AD pathophysiology, but these persons have not yet reached the threshold for a clinical AD diagnosis. As a result, the magnitude of the association can be diluted due to the discordance between AD neuropathology and diagnostic status. For example, in the study outlined above, we found that *APOE* ε4 was associated with AD pathology among persons without dementia, i.e., in analyses restricted to the control group of a case–control study [79•]. This issue is further complicated by individual differences in cognitive or neural reserve [82]. Both structural [83] and neuropsychological [84] components of reserve have been shown to influence the level of resilience in the face of accumulating disease pathology, such that a greater reserve capacity reduces the deleterious effect of AD pathology on clinical symptoms. Without directly assessing genetic influences on disease pathology, such influences could be easily masked by the modifying effect of reserve. In a recent GWAS of AD pathology, Kramer et al. [85•] discovered that polymorphisms in *RELN* were associated with higher burden of neurofibrillary tangles (NFT) among older persons without dementia, and they hypothesized a potential role of reelin in tau phosphorylation and that upregulation of reelin may be a compensatory remedy to tau-related stress.

Third, a compelling rationale for using an endophenotype is to refine and partition a generic phenotype into ones that are associated with very specialized pathways [6]. The fine-tuning helps to address the complexity of the disease biology and can amplify the association of contributory loci along the targeted pathway. Substantial evidence shows that AD tends to be co-existent with other brain lesions like cerebrovascular infarctions, Lewy bodies, and TDP-43, suggesting that not all AD-associated alleles will work through the pathologic accumulation of Aβ and phosphorylation of tau, the pathologic hallmarks that characterize AD. The disease also involves many other biological processes such as oxidative stress [86], chronic inflammation [87], alteration in lipid metabolism [88] and depletion of molecular chaperones [89]. It is essential to disentangle distinct genetic risk factors for the different intermediate traits in order to understand the underlying biological mechanisms that contribute to the onset of AD. Sleegers et al. [90] presented a conceptual model for the implications of recently discovered loci on AD susceptibility highlighting the influence of these novel loci on different aspects of AD pathophysiology. Clusterin (*CLU*) was hypothesized to share many properties of *APOE* in regulating Aβ formation and lipid transportation; complement receptor 1 (*CR1*) on the other hand likely contributed to chronic inflammation and C3b-mediated clearance; and phosphatidylinositol binding clathrin assembly protein (*PICALM*) was implicated in maintaining synaptic function and mediating endocytosis in *APP* recycling.

Pathologic phenotypes offer the promise of assessing different mechanistic hypotheses of action for each risk allele. For example, we showed that *CR*1, but not *CLU* and *PICALM,* was significantly associated with deposition of neuritic plaques, and this association further mediates, in part, the effect of the *CR1* locus on cognitive decline [91•]. However, the *CR1* locus also affects the accumulation of cerebral amyloid angiopathy [92] and may therefore also function through an effect on the cerebral vasculature. Beyond demonstrating the utility of leveraging intermediate phenotypes to build a causal chain of events leading from a risk factor to a clinical syndrome, *CR1* also illustrates the strategy of using an intermediate phenotype to perform fine mapping of a susceptibility locus, which can help to locate the causal variant and to find additional variants that have an effect on AD and its pathology [93]. These types of studies are not unique to *APOE* and *CR1*, as evidence supporting an association of the *CETP* AD susceptibility allele with AD pathology has recently been reported [94]. Further, an interesting study from Brazil reported that an individual’s proportion of African ancestry was associated with a lower burden of neuritic plaque pathology, although no specific variants were reported [95].

## Cognition Endophenotype

In parallel to the neuropathologic phenotypes, cognitive endophenotypes (i.e., level of cognitive function and rate of decline in cognition) serve as another promising alternative in gene discovery [96]. These quantitative measures share the strengths of the neuropathologic phenotypes as presented above. In particular, they are independent of diagnostic status and can be assessed in persons with and without the clinical manifestation of the disease, which helps to overcome the obstacle of confounding due to pre- and sub-clinical contamination in the control group. We and others have shown that cognitive decline begins years prior to a clinical diagnosis of AD or MCI [97]. Further, because of their quantitative nature, statistical power to capture heritable variation is improved. It is now clear that a better understanding of the earlier stage in the disease progression holds great promise for effective prevention and intervention strategies, and cognitive phenotypes can help to detect genetic risk factors attributable to the preclinical and subclinical change in cognition that are not likely to be captured in conventional case–control studies.

An important additional strength of cognitive endophenotypes that complements neuropathologic traits is that they can be measured within the individuals longitudinally throughout life. Compared with cross-sectional data, these longitudinal data directly address the question of change over time [98]. AD is the result of a sequence of pathophysiological events from Aβ deposition to synaptic dysfunction, to tangle formation, to other structural changes [99••]. Trajectories of change in cognition characterized by repeated assessments of cognition provide objective evidence about how AD manifests over time. Endophenotypes such as cognitive decline have been increasingly used [91•, 93, 94, 96, 100•, 101–107] to explore the genetic linkage to these trajectories in two important ways.

First, the AD loci discovered so far in cross-sectional susceptibility studies are interestingly not in concordance with those that account for the disease progression. In a recent genome-wide scan, none of the known AD susceptibility variants, except *APOE* and *CR1*, were found to be significantly associated with the rate of cognitive decline [100•]. On the other hand, the study reported a highly suggestive association with a locus near the *PDE7A* and *MTFR1* genes which regulate inflammation and oxidative stress, respectively. Similar non-findings were reported by a separate GWAS effort, where minor allele homozygosity of multiple novel variants were found to be associated with a faster rate of disease progression in subjects with mild cognitive impairment, but none of these variants matched those identified in previous susceptibility studies [107]. However, it should be noted that the sample sizes of these cognitive decline GWAS are a fraction of the size of the case/control studies, so it is too early to make definitive statements about known and novel susceptibility loci. Estimates suggest that >5,000 subjects will be needed to begin to have reasonable power to identify a given variant [100•].

Second, AD develops slowly over decades and the cognitive trajectories cover a wide spectrum from preclinical phase of AD [108] all the way through the terminal decline in the last few years of life [109]. Specific AD susceptibility loci could be associated with different aspects of this cascade; therefore, it is plausible that they differentially affect various stages of the cognitive trajectory. Intermediate phenotypes like cognitive decline provide additional utility in dissecting the functional pathway in gene action. *APOE* is again illustrative on this point: the AD susceptibility allele *APOE* ε4 was discovered decades ago, and so far little is known regarding where the polymorphism exerts its effect over the course of the disease. In particular, it is not clear whether the effect of the *APOE* locus persists after the onset of dementia or whether it differs in its magnitude of influence along the progression. Most literature consistently reports the effect of ε4 on the risk of incident AD [110–113] and decline in cognitive performance in persons free of dementia [102, 114–117, 118•, 119, 120]. Controversy arises on whether there is an ε4 effect on cognitive decline in the late stages of the disease. Some studies suggest that ε4 is not related to decline after a diagnosis of AD [121–125], which would support the theory that *APOE* works primarily as a triggering factor [126]. On the other hand, other studies have found that the ε4 allele remains as an important predictor of the progression to AD after subjects experience cognitive impairment [127, 128] and is associated with cognitive decline in the early stages of AD [129]. To unravel these controversies, more complex analyses such as nonlinear mixed models could be considered for studies that have a sufficient number of cognitive evaluations over a long enough time [130]. Recently, using random change point models, we have showed that, among participants who were dementia free at enrollment but later developed incident AD, ε4 carriers had a more rapid cognitive decline both before and after the onset of AD dementia [80]. The capacity to incorporate these types of analyses into high-throughput gene discovery programs will best exploit the use of endophenotypes derived from longitudinal data for GWAS.

## Future Directions

**Fig. 2:**
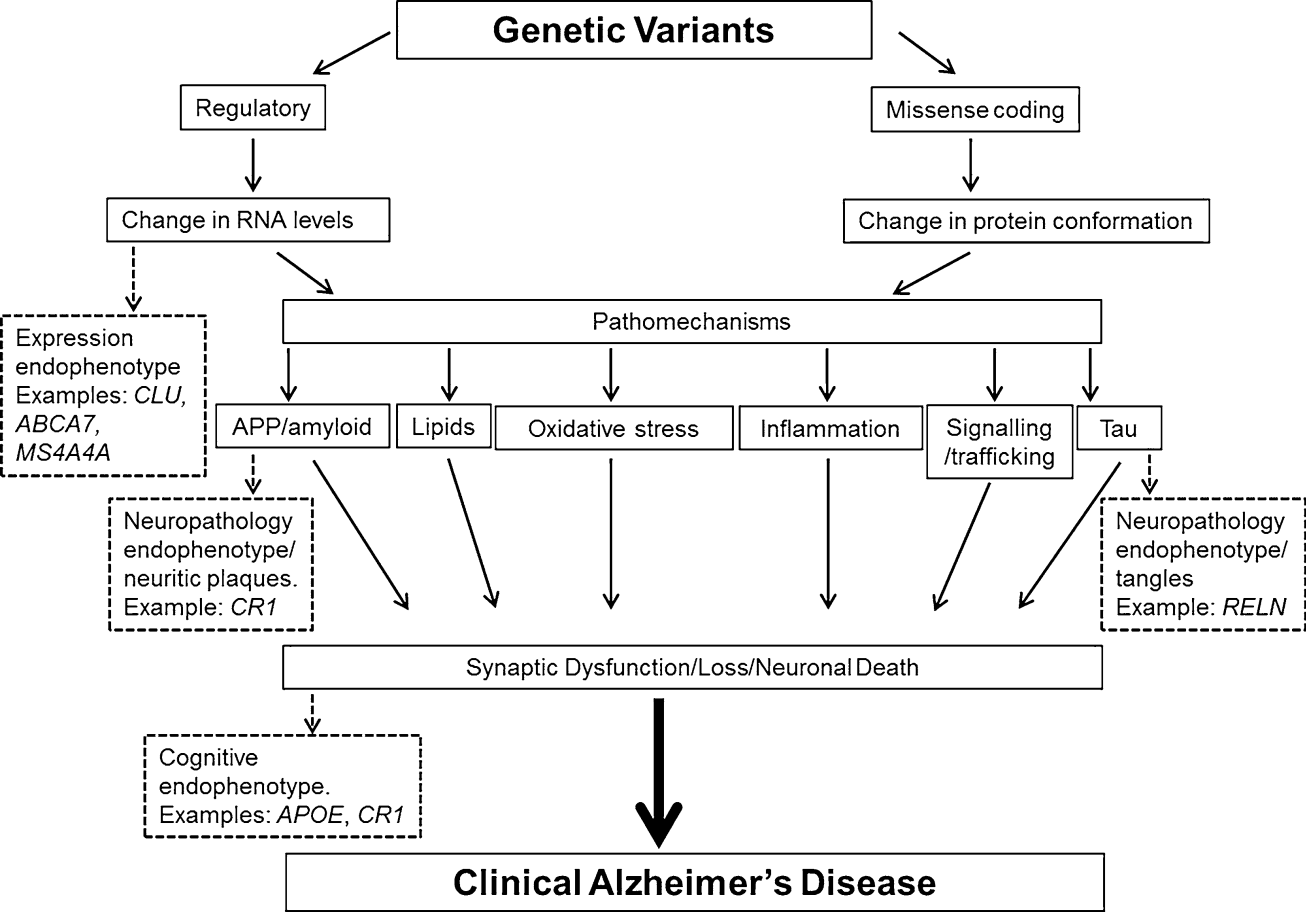
Simplified model for the pathophysiology of AD and its endophenotypes: the flow of major pathogenic mechanisms from *top* to *bottom* represent the proposed consequence of events. The interactions between the various pathomechanisms are omitted for simplicity. The *dotted arrows* and *boxes* symbolize the pathogenic events that presumably precede the endophenotypes, and the endophenotypes, respectively. The examples of genes that are associated with the endophenotypes are taken from the text. While there are clearly many other plausible endophenotypes in this cascade, only those that are the focus of this review are shown

As the meta-analyses of AD GWAS come to a close, we will have a number of validated and suggested susceptibility loci whose functional consequences can begin to be elucidated by leveraging pertinent intermediate traits such as the ones that we have discussed: RNA expression, neuropathologic measures and cognitive measures (Fig. 2). As illustrated by the *APOE* and *CR1* loci, such studies can be critical in tying risk factors to a particular aspect of AD-related pathophysiology and can lead to the elaboration of a causal chain of events linking risk factors to a clinical syndrome such as AD. Furthermore, gene expression studies in combination with disease association can nominate transcriptional regulatory mechanisms as a testable culprit for novel AD GWAS loci, such as *CLU*, *ABCA7* and *MS4A4A*, as discussed. However, beyond gene discovery, the AD GWAS have had an added benefit in that the genotyping they have performed included many thousands of subjects with pertinent intermediate traits, and these genotype data can now be repurposed for discovery studies targeting the intermediate traits [34•, 56•, 100•]. A major limitation of such efforts is the fact that, in many cases, the intermediate phenotypes were not collected systematically in the same manner or using the same strategy across different cohort studies, which complicates the merging of results across individual studies and reduces our statistical power. Nonetheless, such meta-analyses for gene expression, neuropathologic, cognitive and other traits is clearly an important goal for the near future as large sample sizes will be needed for these studies, as has been well demonstrated by the case/control approaches. While repurposing existing data is a valuable activity that will yield insights, we ultimately need to gather as many intermediate traits as possible from the same subjects, as this allows us to fully explore the relationship of these traits and how the effect of a risk factor (genetic or environmental) propagates to influence AD susceptibility. The AD neuroimaging initiative [131] and prospective cohort studies of aging, such as the Mayo Clinic Study of Aging [22], and Religious Order Study and the Memory and Aging Project, which also have brain donations [74], are excellent illustrations of the type of resource that are needed to powerfully investigate the pathophysiology of AD and other neuropsychiatric diseases. Such studies, if they were tenfold larger, would provide ideal platforms for communities of investigators in this field to explore the chain of events linking risk factors to a clinical syndrome. This information will be critical in the successful translation of gene discoveries to viable therapeutic approaches.
